# Mortality of *Candida auris* candidemia compared with non-*auris* candidemia: a systematic review and meta-analysis

**DOI:** 10.3389/fcimb.2026.1952600

**Published:** 2026-10-02

**Authors:** Jing Chen, Na Xiao, Qian Xiang, Yan Luo, Hui Wang, Tian-fen Liao

**Affiliations:** Department of Healthcare-Associated Infection Control Center, Sichuan Provincial People’s Hospital, University of Electronic Science and Technology of China, Chengdu, China

**Keywords:** bloodstream infection (BSI), *Candida auris*, candidemia, meta-analysis, mortality

## Abstract

**Background:**

*Candida auris* has emerged as a globally significant healthcare-associated fungal pathogen. Although descriptive studies have reported high mortality for candidemia caused by *C. auris*, whether the mortality differs from non-*auris* candidemia remains unclear. We conducted this meta-analysis to compare the mortality between patients with *C. auris* and non-*auris* candidemia.

**Methods:**

We searched PubMed (including MEDLINE), Embase, and Web of Science databases for relevant studies published before June 2026. A random-effects model was used for meta-analysis.

**Results:**

A total of 16 articles were included in this meta-analysis. In the pooled crude analysis, mortality was significantly lower in patients with *C. auris* candidemia than in those with non-*auris* candidemia (OR = 0.77, 95% CI 0.67-0.90). Subgroup analyses showed significant geographic variation (P for interaction = 0.019), with lower mortality observed in studies from Turkey, the Middle East and the Americas, but not in South Asia and Africa. Despite lower mortality, *C. auris* candidemia was associated with prolonged hospitalization in descriptive analysis.

**Conclusion:**

In this meta-analysis of crude estimates, mortality was significantly lower in patients with *C. auris* candidemia than in those with non-*auris* candidemia. However, prolonged hospitalization in *C. auris* infection highlights its medical resource burden. For clinicians, management of *C. auris* candidemia should focus on timely diagnosis, appropriate antifungal therapy, and strengthened infection prevention, especially in cases of long-term hospitalization.

## Introduction

Candidemia remains a common healthcare-associated infection that can lead to poor prognosis, prolonged hospitalization, and increased medical costs, and has become a global public health concern (Magill et al., 2014). Despite advances in antifungal therapy, the mortality rate of candidemia remains high, overall exceeding 30% and reaching up to 40% in intensive care unit (ICU) patients (Kang et al., 2017; Koehler et al., 2019). The risk of developing candidemia is closely tied to healthcare exposure and impaired host defenses, with broad-spectrum antibacterial use, central venous catheters, major surgery, total parenteral nutrition and prolonged ICU stay considered as risk factors (Kullberg and Arendrup, 2015; Pappas et al., 2018). Once candidemia develops, advanced age, greater illness severity, and delayed or inadequate antifungal therapy are associated with severe disease and increased mortality (Pappas et al., 2018). In the past, *Candida albicans* was the main pathogen of candidemia. However, the epidemiological characteristics seem to have changed in recent years, with an increasing incidence of invasive infections caused by *non-albicans Candida* species, which urgently needs attention (Deorukhkar et al., 2014; da Matta et al., 2017; Schroeder et al., 2020; Kitaya et al., 2023). In this case, *Candida auris* becomes an emerging pathogen that can cause healthcare-associated infections. Since its first identification in 2009, *Candida auris* has caused multiple outbreaks globally and has become a major public health concern due to its multidrug resistance and high transmissibility (Satoh et al., 2009; Schelenz et al., 2016; Mulet Bayona et al., 2020; Liu et al., 2024; Cornely et al., 2025). For example, a persistent intensive care unit outbreak in the United Kingdom from 2015 to 2017 was terminated only after removal of the temperature probes, and a European tertiary care hospital reported 41 invasive candidaemia during 2016-2017 (Eyre et al., 2018; Ruiz-Gaitan et al., 2018). Unlike other *Candida* species, *C. auris* exhibits strong capacity for long-term environmental survival, persistent skin colonization, biofilm formation, and resistance to routine disinfection, thereby promoting nosocomial transmission (Sherry et al., 2017; Welsh et al., 2017; Forsberg et al., 2019). Patients can remain colonized with *C. auris* for weeks to months or longer, often without symptoms (Preventing the Spread of C. auris). This asymptomatic carriage facilitates silent spread within healthcare settings and complicates outbreak control efforts. In 2022, the World Health Organization (WHO) listed *C. auris* as a priority fungal pathogen of concern (Parums, 2022).

*C. auris* infection poses a challenge to clinicians due to its difficulties in laboratory identification, antifungal resistance, and ability of environmental persistence and transmission (Welsh et al., 2017). A meta-analysis of cases from different countries between 2009 and 2019 reported that a crude mortality rate of 45% for *C. auris* candidemia, indicating a high mortality burden in candidemia caused by *C. auris* (Chen et al., 2020). However, it remains unclear whether mortality associated with *C. auris* candidemia is higher than that of candidemia caused by non-*auris Candida* species. Some studies have reported significantly lower 30-day mortality in patients with *C. auris* candidemia compared to those with non-*auris* candidemia (Ortiz-Roa et al., 2023; Arslan et al., 2026), while others have found mortality rates comparable to those of candidemia caused by non-auris *Candida* species (Simon et al., 2023; Gulten et al., 2025). This inconsistency may stem from differences in population characteristics, geographic regions, healthcare settings, medical conditions, and antifungal treatment strategies. Beyond mortality, previous studies have reported several clinical differences between *C. auris* and non-*auris Candida* candidemia. Patients with *C. auris* infection usually have longer hospital stay before infection, higher rates of prior antifungal exposure, higher rates of mechanical ventilation and urinary catheter use (Alvarez-Moreno et al., 2023; Athavale-Wad et al., 2025; Gulten et al., 2025; Arslan et al., 2026). To date, whether *C. auris* candidemia has a different risk of death compared to other *Candida* species remains unresolved.

Therefore, we conducted a meta-analysis to compare the mortality rates between patients with *C. auris* and non-*auris Candida* candidemia. The primary objective was to compare the mortality rates of candidemia caused by *C. auris* and non-*auris Candida*, and the secondary objective was to evaluate the difference in duration of hospitalization between the two groups.

## Methods

### Search strategy

We systematically searched PubMed (including MEDLINE), Embase, and Web of Science databases for relevant studies up to June 30, 2026. The following keywords were combined to search for literature: (*Candida auris* or *C. auris*) and (candidemia or bloodstream infection or fungemia) and (mortality or survival or outcome). The complete search strategies are provided in **Supplementary File S1**. All identified records were compiled and duplicate records were removed manually before screening. We also scanned the reference lists of the paper to identify additional studies. Two authors independently reviewed these studies, and any disagreements were resolved through discussion and consensus. This review was not prospectively registered on PROSPERO, as a similar protocol had already been registered at the time we attempted to register. Nonetheless, we followed the PRISMA 2020 guidelines to ensure methodological transparency, and the completed PRISMA checklist was provided as **Supplementary File S2**.

### Study selection

Two independent reviewers screened the titles and abstracts of the retrieved search records based on inclusion and exclusion criteria, and then reviewed the full texts of potential eligible articles to confirm their eligibility. The third reviewer resolved any disagreements. Studies were included if they met the following criteria based on the PICOS framework: Population (P): Hospitalized patients diagnosed with microbiologically confirmed candidemia, defined as at least one positive blood culture for *Candida* species. Intervention/Exposure (I): Candidemia caused by *Candida auris*. Comparator (C): Candidemia caused by *non-auris Candida* species, including but not limited to *C. albicans*, *C. glabrata*, *C. parapsilosis*, and *C. tropicalis*. Outcome (O): The primary outcome was all-cause mortality, reported at any measurable time point (e.g., 30-day, 28-day, 90-day, or in-hospital mortality). The secondary outcome was length of hospital stay. Study design (S): Observational studies (retrospective or prospective cohort studies, case-control studies) or randomized controlled trials that provided comparative data between *C. auris* and *non-auris Candida* groups. Studies were excluded if they met one or more of the following criteria: (1) reported only on *C. auris* colonization or infection at non-bloodstream sites without providing candidemia data; (2) included only a single-group design (*C. auris* only) without comparison with *non-auris*; (3) case reports, case series with <10 cases per group, reviews, editorials, or conference abstracts; (4) without sufficient data for analysis; (5) non-English published studies.

### Data extraction

Data were extracted independently and cross-checked by two reviewers. Disagreements between the two reviewers were resolved through discussion. If consensus could not be reached, a third reviewer was consulted. The following information was extracted: first author, publication year, the country where the study was conducted, study design, clinical setting, study period, mortality rate of candidemia by *C. auris* and non-*auris*, and the definition of outcome. When feasible, corresponding authors were contacted for missing or ambiguous data.

### Quality assessment

We assessed the methodological quality of all included studies using the Newcastle Ottawa scale (NOS) for the quality assessment of cohort studies and case–control studies (Stang, 2010). The total score of each article was the sum of all items assessed as positive. Studies were categorized as high quality (7–9 points), moderate quality (4–6 points) and low quality (1–3 points) based on the score.

### Statistical analysis

All statistical analyses were performed with Statistical Software-Stata, version 15.0. Odds ratios (ORs) with 95% confidence intervals (CIs) were used as the primary effect measure for all-cause mortality. Crude ORs were used as the primary effect measure because adjusted estimates were not uniformly reported across studies, and using crude ORs ensured a consistent analytical approach and maximized the number of studies available for the primary analysis. Adjusted estimates, where reported, were not pooled due to the heterogeneity in effect measures and adjusted covariates. Crude ORs were derived from 2×2 tables for each study, using the number of events and total patients in each group (*C. auris* vs. *non-auris*). For studies that did not report crude event counts but presented Kaplan-Meier curves, mortality data were reconstructed using the method described by Tierney et al. (2007), which extracts time-to-event data from published survival curves. When multiple time points were reported, 30-day mortality was preferentially extracted if available; otherwise, the time point closest to 30 days (e.g., 28-day mortality) was used. To evaluate the impact of pooling different time points of mortality, we performed a sensitivity analysis limited to studies reporting 28-day or 30-day mortality. To improve clinical interpretability, we also performed a sensitivity analysis using risk ratios (RR). The statistical heterogeneity among studies was assessed using Cochran’s Q test and I^2^ statistic (Higgins and Thompson, 2002). P value of less than 0.10 for Q statistic and I^2^ greater than 50% was considered as statistically significant heterogeneity. Due to the expected heterogeneity of the included studies, a random-effects model was applied for all analyses. We performed subgroup analysis according to study setting (ICU-only vs. mixed hospital population) and geographic region (Turkey/Middle East vs. Americas vs. South Asia vs. Africa). Funnel plots and Egger regression test were used to evaluate publication bias, and a P value of less than 0.1 was considered indicative of significant publication bias.

## Results

### Search results

We identified 282 non-duplicate articles in the search. Following title and abstract screening, 29 articles were reviewed for full text. Ultimately, 16 articles were considered eligible and included for further analysis. The literature screening process was shown in **Figure 1**.

**Figure 1:**
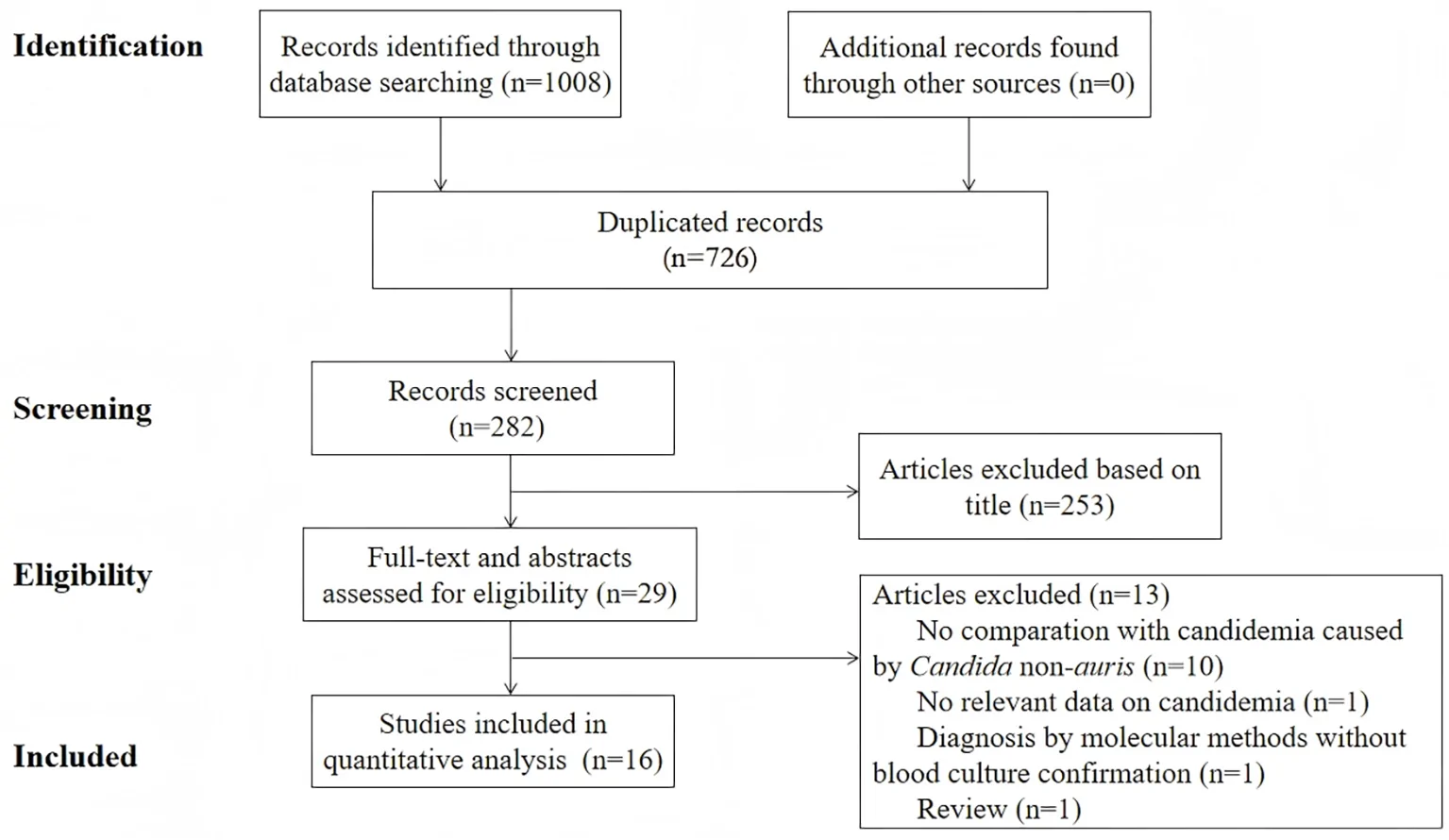
Screening and selection process of studies.

### Study characteristics

The sixteen included studies were published between 2019 and 2026 (van Schalkwyk et al., 2019; Caceres et al., 2020; Sayeed et al., 2020; Moin et al., 2021; Parak et al., 2022; Alvarez-Moreno et al., 2023; Ortiz-Roa et al., 2023; Simon et al., 2023; Athavale-Wad et al., 2025; Chaari et al., 2025; Choudhury et al., 2025; Gulten et al., 2025; Arslan et al., 2026; Korkusuz et al., 2026; Park et al., 2026; Perez et al., 2026). The characteristics of the included studies were summarized in **Table 1**. Among these studies, three were conducted in Turkey (Gulten et al., 2025; Arslan et al., 2026; Korkusuz et al., 2026), three in Colombia (Caceres et al., 2020; Alvarez-Moreno et al., 2023; Ortiz-Roa et al., 2023), two in the United States (Simon et al., 2023; Perez et al., 2026), two in Pakistan (Sayeed et al., 2020; Moin et al., 2021), two in India (Athavale-Wad et al., 2025; Choudhury et al., 2025), two in South Africa (van Schalkwyk et al., 2019; Parak et al., 2022), one in Bahrain (Chaari et al., 2025) and one in Northern Emirates (Park et al., 2026). Among the included studies, 7 were multi-center studies (van Schalkwyk et al., 2019; Caceres et al., 2020; Alvarez-Moreno et al., 2023; Ortiz-Roa et al., 2023; Simon et al., 2023; Chaari et al., 2025; Perez et al., 2026), and the remaining 9 were single-center studies (Sayeed et al., 2020; Moin et al., 2021; Parak et al., 2022; Athavale-Wad et al., 2025; Choudhury et al., 2025; Gulten et al., 2025; Arslan et al., 2026; Korkusuz et al., 2026; Park et al., 2026). In terms of study design, 9 were cohort studies (van Schalkwyk et al., 2019; Sayeed et al., 2020; Moin et al., 2021; Ortiz-Roa et al., 2023; Chaari et al., 2025; Choudhury et al., 2025; Korkusuz et al., 2026; Park et al., 2026; Perez et al., 2026) and 7 were case-control studies (Caceres et al., 2020; Parak et al., 2022; Alvarez-Moreno et al., 2023; Simon et al., 2023; Athavale-Wad et al., 2025; Gulten et al., 2025; Arslan et al., 2026). The sixteen studies included a total of 16525 patients with candidemia, comprising 1409 cases of *C. auris* and 15116 cases of *non-*auris. Regarding mortality, ten studies reported 30-day mortality directly (Caceres et al., 2020; Sayeed et al., 2020; Parak et al., 2022; Alvarez-Moreno et al., 2023; Ortiz-Roa et al., 2023; Simon et al., 2023; Gulten et al., 2025; Arslan et al., 2026; Korkusuz et al., 2026; Perez et al., 2026); one additional study reported 30-day Kaplan-Meier estimates (Park et al., 2026), from which mortality data were reconstructed using the Tierney method. In addition, two studies reported 28-day mortality (Athavale-Wad et al., 2025; Chaari et al., 2025), two reported in-hospital mortality (van Schalkwyk et al., 2019; Moin et al., 2021), and one reported mortality during ICU stay (Choudhury et al., 2025).

**Table 1:** Characteristics of included studies.

Author, year	Country	Study design	Clinical setting	Study period (year)	Mortality rate (*Candida auris*/non-*auris*)	Mortality definition
(Simon et al., 2023)	USA	Multicenter retrospective case-control	Mixed	2016-2020	30.1%/38.9%	30-day
(Arslan et al., 2026)	Turkey	Single-center retrospective case-control	ICU	2021-2023	63.3%/78.4%	30-day
(Moin et al., 2021)	Pakistan	Single-center retrospective cohort	Mixed	2020	66.7%/65.0%	In-hospital
(Athavale-Wad et al., 2025)	India	Single-center retrospective case–case study	Mixed	2019-2024	36.5%/40.1%	28-day
(Sayeed et al., 2020)	Pakistan	Single-center retrospective cohort	Mixed	2014-2017	62.2%/52.5%	30-day
(Gulten et al., 2025)	Turkey	Single-center retrospective case-control	ICU	2022-2024	54.5%/69.1%	30-day
(Korkusuz et al., 2026)	Turkey	Single-center retrospective cohort	Mixed	2021-2024	48.4%/60.2%	30-day
(Ortiz-Roa et al., 2023)	Colombia	Multicenter retrospective cohort	Mixed	2016-2021	38.1%/51.1%	30-day
(Alvarez-Moreno et al., 2023)	Colombia	Multicenter prospective case–case study	Mixed	2016–2017	31.8%/40.4%	30-day
(Park et al., 2026)	Northern Emirates	Single-center retrospective cohort	Mixed	2019-2025	36.7%/37.5%	30-day
(Parak et al., 2022)	South Africa	Single-center retrospective case-control	Mixed	2015-2018	42.2%/44.4%	30-day
(van Schalkwyk et al., 2019)	South Africa	Multicenter retrospective surveillance	Mixed	2016-2017	45.1%/42.5%	In-hospital
(Choudhury et al., 2025)	India	Single-center retrospective cohort	ICU	2020-2023	31.6%/34.5%	ICU mortality
(Perez et al., 2026)	USA	Multicenter retrospective cohort	Mixed	2015-2024	29.6%/32.5%	30-day
(Chaari et al., 2025)	Bahrain	Multicenter retrospective cohort	ICU	2022-2024	66.7%/87.5%	28-day
(Caceres et al., 2020)	Colombia	Multicenter retrospective case-case study	ICU	2015-2016	42.5%/40.0%	30-day

### Study quality

Among the included studies, twelve studies had high quality (van Schalkwyk et al., 2019; Caceres et al., 2020; Parak et al., 2022; Ortiz-Roa et al., 2023; Simon et al., 2023; Athavale-Wad et al., 2025; Chaari et al., 2025; Gulten et al., 2025; Arslan et al., 2026; Korkusuz et al., 2026; Park et al., 2026; Perez et al., 2026), while the remaining four studies had moderate quality (Sayeed et al., 2020; Moin et al., 2021; Alvarez-Moreno et al., 2023; Choudhury et al., 2025)(**Supplementary Figure S1**).

### Overall mortality

In the pooled crude analysis of all 16 studies, the mortality rate was significantly lower in the *C. auris* group compared with the *non-auris* group (OR = 0.77, 95% CI 0.67-0.90; I^2^ = 18.2%), with a low heterogeneity among these studies (**Figure 2**). In the sensitivity analysis limited to the 13 studies reporting 28-day or 30-day mortality, the pooled OR was 0.75 (95% CI: 0.65-0.87), consistent with the primary analysis and confirming the robustness of the results. A sensitivity analysis using RR yielded a pooled RR of 0.87 (95% CI 0.81-0.93), consistent with the primary OR estimate. At the individual study level, three studies reported significantly lower mortality in the *C. auris* group (Ortiz-Roa et al., 2023; Arslan et al., 2026; Korkusuz et al., 2026), while the remaining thirteen studies showed non-significant trends toward lower mortality or comparable mortality between groups (van Schalkwyk et al., 2019; Caceres et al., 2020; Sayeed et al., 2020; Moin et al., 2021; Parak et al., 2022; Alvarez-Moreno et al., 2023; Simon et al., 2023; Athavale-Wad et al., 2025; Chaari et al., 2025; Choudhury et al., 2025; Gulten et al., 2025; Park et al., 2026; Perez et al., 2026). No study reported a significant increase of mortality in the *C. auris* group.

**Figure 2:**
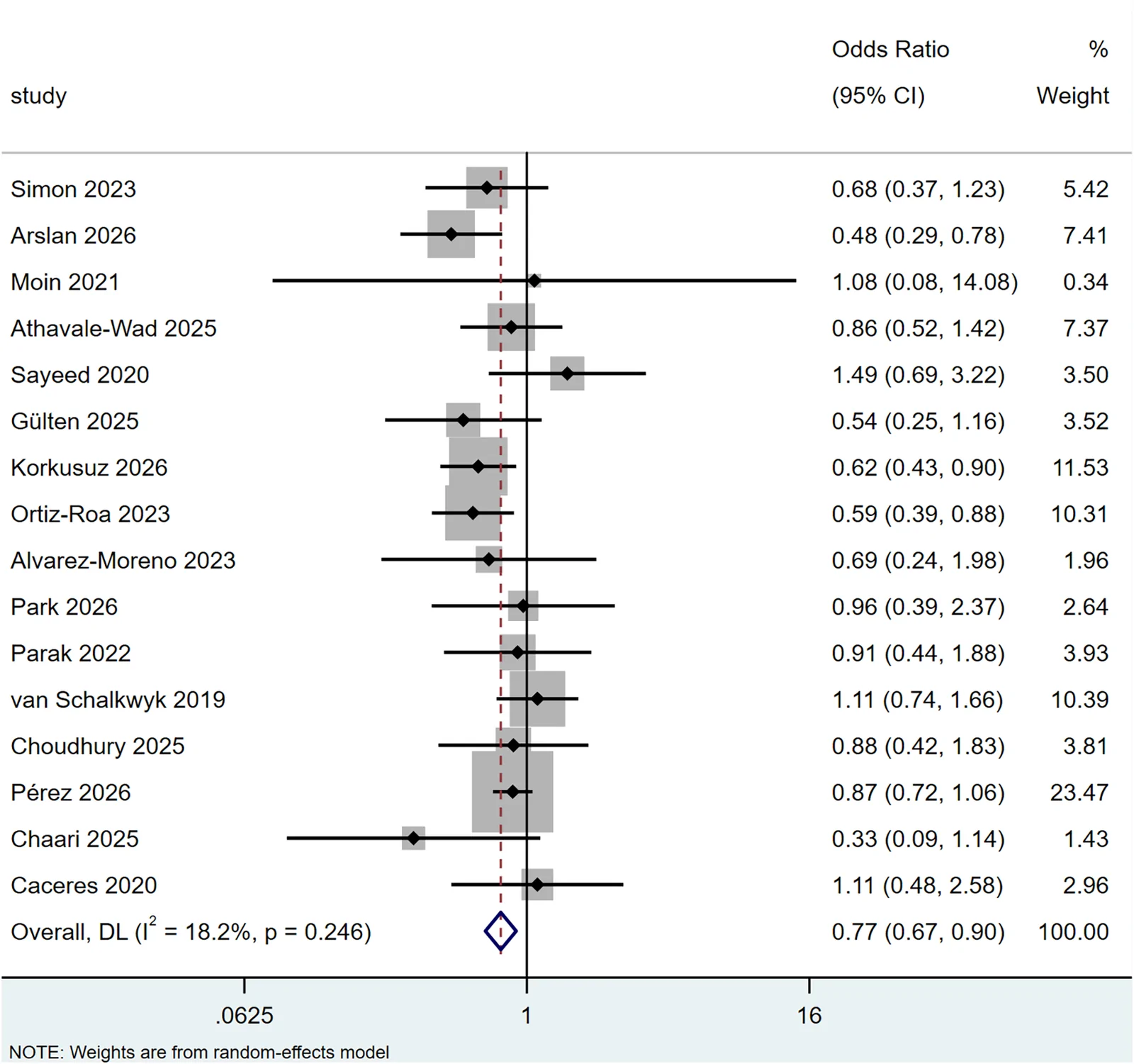
Forest plot for mortality in *C. auris* vs. non-*auris* candidemia.

### Subgroup analyses

Subgroup analyses were conducted by study setting and geographic region to explore potential sources of heterogeneity. According to the study setting, both ICU-only studies (k = 5; OR = 0.61, 95% CI 0.42-0.89) and mixed hospital population studies (k = 11; OR = 0.82, 95% CI 0.72-0.94) showed lower mortality in the *C.* auris group, with no significant difference between groups (P = 0.142) (**Figure 3**). The heterogeneity of both subgroups was low (I^2^ = 0%).

**Figure 3:**
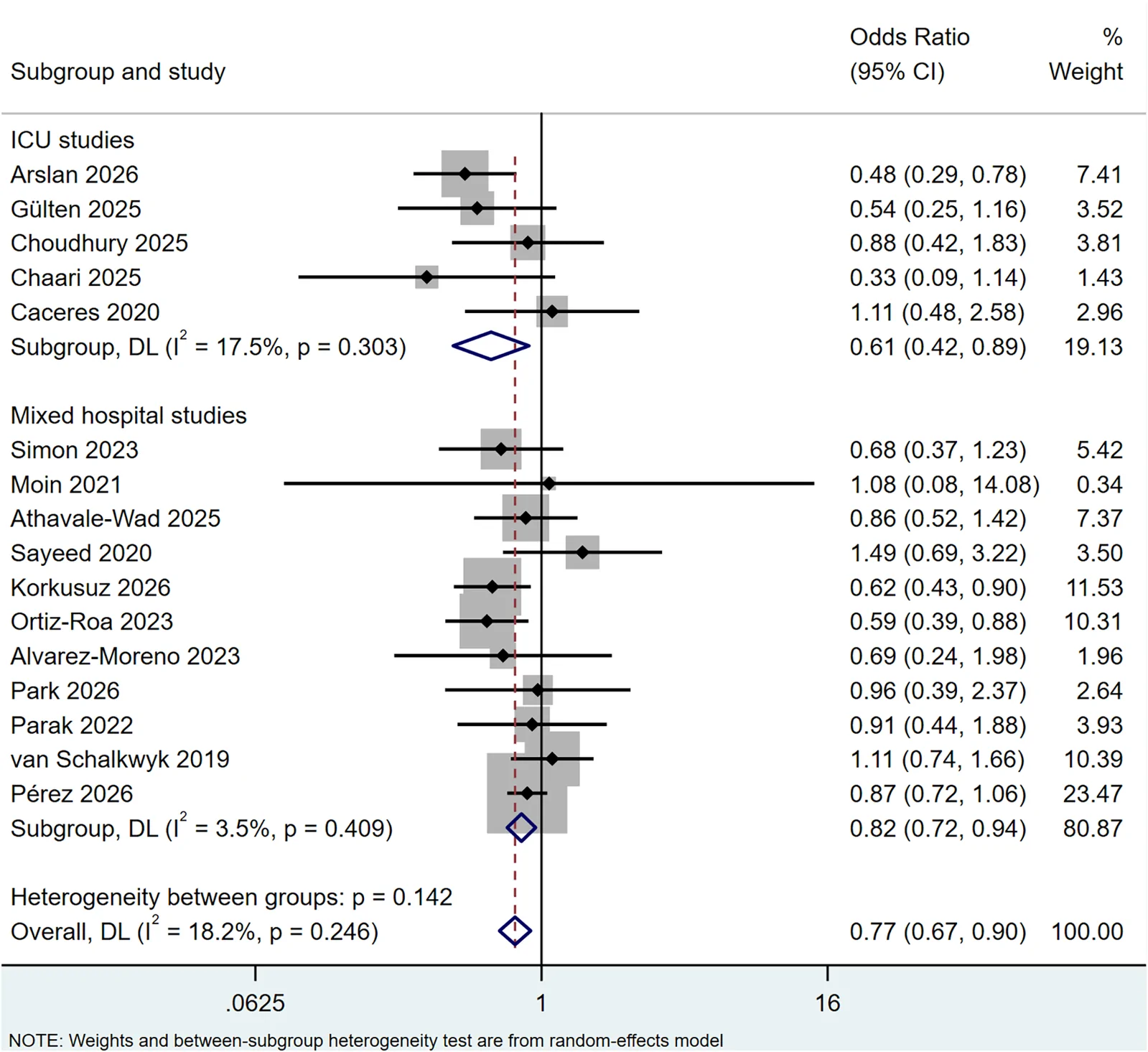
Forest plot of subgroup analysis for mortality in *C. auris* vs. non-*auris* candidemia stratified by study setting.

Geographic differences were observed. The mortality rate of *C. auris* group in Turkey/Middle East (k = 5; OR = 0.57, 95% CI 0.44-0.74) and the Americas (k = 5; OR = 0.80, 95% CI 0.68-0.95) was significantly lower, but there was no significant difference in South Asia (k = 4; OR = 0.98, 95% CI 0.68-1.40) and Africa (k = 2; OR = 1.06, 95% CI 0.75-1.50) (**Figure 4**). The test for subgroup difference was statistically significant (P = 0.019), and the heterogeneity of all regional subgroups was low (I^2^ = 0%).

**Figure 4:**
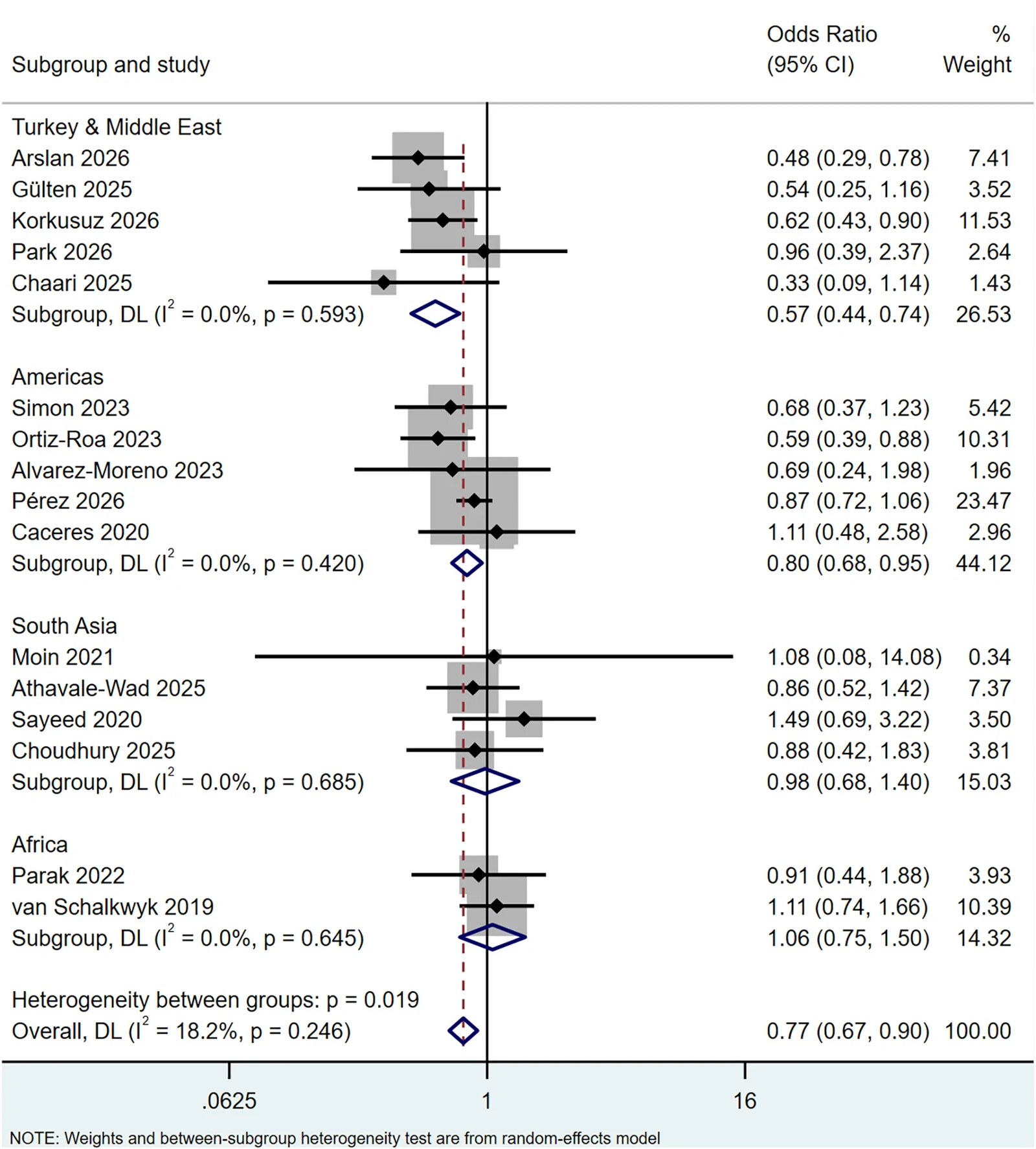
Forest plot of subgroup analysis for mortality in *C. auris* vs. non-*auris* candidemia stratified by geographic region.

### Publication bias

Publication bias was assessed using the Egger regression test, and no publication bias was found in the analysis (P = 0.634).

### Length of hospital stay

Seven studies reported comparative data on length of hospital stay (LOS), including four in mixed hospital settings (total LOS) (van Schalkwyk et al., 2019; Sayeed et al., 2020; Parak et al., 2022; Ortiz-Roa et al., 2023) and three in ICU-only settings (ICU LOS) (Chaari et al., 2025; Choudhury et al., 2025; Arslan et al., 2026).Due to the difference in reporting methods of LOS, no quantitative summary analysis was conducted for this outcome. Among four studies reporting total LOS, three demonstrated significantly longer hospitalization in the *C. auris* group (van Schalkwyk et al., 2019; Sayeed et al., 2020; Parak et al., 2022), while one showed no significant difference (Ortiz-Roa et al., 2023). Three studies conducted only in ICU showed longer ICU stays in the *C.auris* group, with two of them reaching statistical significance (Chaari et al., 2025; Arslan et al., 2026). Overall, the above studies suggested a trend toward prolonged hospitalization in the *C. auris* group, although the results should be interpreted with caution.

## Discussion

In this meta-analysis, we included 16 comparative studies involving 16,525 patients from 8 countries. The pooled crude analysis showed that the mortality was significantly lower in patients with *C. auris* candidemia than in those with non-*auris* candidemia, with low heterogeneity. This finding provides updated and quantified evidence on the comparative mortality of *C. auris* and non-*auris* candidemia.

These findings challenge the prevailing perception that *C. auris* carries a higher mortality risk than other *Candida* species. Previous systematic review reported that *C. auris* candidemia had a high crude mortality rate, but no comparative analysis had been conducted with non-*auris* species (Chen et al., 2020; Kim et al., 2024). Our study addresses this gap by pooling comparative studies and provides the first quantitative estimate of the relative mortality effect. In the crude analysis, the *C. auris* group had lower mortality than the non-*auris* group. Several possible mechanisms may explain the lower mortality rate observed in the *C. auris* group. Firstly, the increased clinical awareness of antifungal drug resistance and outbreak potential of *C. auris* may lead to earlier suspicion, faster diagnostic evaluation, and timely initiation of empirical antifungal therapy. Secondly, the detection of *C. auris* often triggers more active clinical management, including stricter contact precautions and enhanced environmental disinfection, which may indirectly improve patient prognosis. Thirdly, comparative animal models have suggested that *C. auris* may have lower virulence than *C. albicans* (Du et al., 2020). This reduced virulence is attributed to the inability of *C. auris* to form hyphae or pseudohyphae, which play an important role in invading tissues during infection (Yue et al., 2018).

Subgroup analysis showed that both ICU-only and mixed hospital populations studies had lower mortality in the *C. auris* group compared with the non-*auris* group, with no significant between-group difference. This consistency supports the robustness of our primary findings and suggests that the lower mortality observed in the *C.auris* group may not be limited to specific clinical environments. Another subgroup analysis observed significant geographical differences. In the crude analysis, the mortality rate of *C. auris* candidemia in Turkey/Middle East and the Americas was significantly lower than that of non-*auri*s, while there was no significant difference in South Asia and Africa. This difference may reflect differences in epidemiological background, healthcare infrastructure, population characteristics, and the distribution of phylogenetic clades. Currently, *C. auris* contains six major phylogenetic clades related to geographic regions, among which Clade 1, 3, and 4 exhibit high levels of antifungal resistance (Lockhart et al., 2017; Forgacs et al., 2020; Suphavilai et al., 2024). Interestingly, South Asia clade (clade I) is the most common clade in Turkey/Middle East and Americas (Hu et al., 2021; Erkose Genc et al., 2024; Parnell et al., 2026). In Turkey and Middle East, where several studies were conducted during institutional outbreaks of *C. auris*, the combination of active surveillance and empiric echinocandin use may have contributed to the observed lower mortality (Erturk Sengel et al., 2023; Alanazi et al., 2025). In South Asia and Africa, the absence of significant differences in mortality rates may reflect limited clinical awareness, inadequate diagnostic ability, and restricted access to effective drugs. However, these interpretations remain speculative and should be confirmed by future studies.

Although mortality was lower with *C. auris* candidemia in the crude analysis, studies that reported length of stay consistently showed prolonged hospitalization. This may reflect the persistence of *C. auris* colonization, the need for prolonged antifungal therapy to achieve microbial clearance, and the time required for implementation of infection control measures before patient discharge, including single-room contact isolation, strict hand hygiene, dedicated medical equipment, active surveillance, and enhanced environmental disinfection. However, this finding should be interpreted with caution, as survivor bias may partly account for the observed longer length of hospital stay-patients who survive longer have the opportunity to be observed for longer hospital stays. Nevertheless, prolonged LOS also has an important impact on healthcare costs and bed resource utilization.

Several limitations of our study should be taken into consideration. Firstly, most of the included studies were retrospective observational studies, and none of them were randomized controlled trials. Although the heterogeneity was low, unmeasured or residual confounding factors could not be excluded. The lack of uniform individual patient-level data precluded adjustment for baseline differences in disease severity, comorbidities (e.g., diabetes mellitus, severe kidney disease, cardiovascular disease, and respiratory diseases), antifungal therapy, and hospital-related factors, the reporting of which varied across studies. In addition, the primary analysis was based on crude ORs, as adjusted effect estimates were not consistently reported across the included studies. Only a few studies reported adjusted estimates, and the covariates used for adjustment varied considerably, making a pooled analysis of adjusted estimates methodologically unreliable. Therefore, our findings represented crude associations rather than independent effects of *C. auris* candidemia on mortality. Secondly, the definition of mortality varied depending on the study (30-day, 28-day, or in-hospital mortality). Although we prioritized 30-day mortality when available, the heterogeneity in outcome definitions might introduce some variability. However, sensitivity analysis limited to 28-day or 30 day mortality yielded consistent results, suggesting that the heterogeneity in mortality definitions did not materially affect the primary findings. Thirdly, the species composition of the non-*auris* comparator groups varied across studies. Furthermore, the non-*auris* control group comprised various *Candida* species, among which *C. albicans*, *C. glabrata*, *C. parapsilosis*, and *C. tropicalis* were the most common. Future large scale, prospective, and multicenter studies are needed to clarify the prognostic differences between *C. auris* and different non-*auris Candida* species.

In conclusion, this meta-analysis suggests that the pooled crude mortality was significantly lower in patients with *C. auris* candidemia compared with those with non-*auris* candidemia. However, this unadjusted association does not imply that *C. auris* candidemia independently has a lower risk of mortality. The prognosis of *C. auris* infection is likely shaped by a complex interplay of host, pathogen, and healthcare system factors. Nevertheless, *C. auris* remains a major public health concern due to its transmissibility, environmental persistence, and multidrug resistance. Clinicians should recognize that for the management of *C. auris*, early suspicion, timely diagnosis, appropriate antifungal treatment, and strengthened infection prevention measures should be emphasized, and prolonged hospitalization and resource utilization should be anticipated. Future research is needed to further validate these findings and clarify the optimal management strategies for *C.auris* candidemia.

## Data Availability

The raw data supporting the conclusions of this article will be made available by the authors, without undue reservation.

## Supplementary material

Supplementary Figure 1 Study quality assessment.

Supplementary File S1 Search Strategies.

Supplementary File S2 PRISMA Checklist.
