# Supplementary material for: Mortality of Candida auris candidemia compared with non-auris candidemia: a systematic review and meta-analysis
Source: Front Cell Infect Microbiol. 2026 Oct 2;16:1952600. doi: 10.3389/fcimb.2026.1952600 (PMC13672011; doi:10.3389/fcimb.2026.1952600)
Supplement: Supplementary file 1 [file Table1.docx]

Complete Search Strategies

Search date: June 30, 2026

1. Pubmed (including MEDLINE)

Platform: PubMed (https://pubmed.ncbi.nlm.nih.gov)

(Candida auris or C. auris) and (candidemia or bloodstream infection or fungemia) and (mortality or survival or outcome)

2. Embase

Platform: Embase (accessed via institutional subscription)

('candida auris' OR 'c auris') AND (candidemia OR 'bloodstream infection' OR fungemia) AND (mortality OR death OR survival OR outcome)

3. Web of science

Platform: Web of science (accessed via institutional subscription)

TS=("Candida auris" OR "C auris") AND (candidemia OR candidaemia OR fungemia OR fungaemia OR "bloodstream infection*") AND (mortality OR survival OR outcome* OR death* OR prognosis)
